# Coffee phytobiome dynamics: integrating multitrophic interactions and plant physiology for climate-resilient production

**DOI:** 10.3389/fmicb.2026.1951767

**Published:** 2026-09-10

**Authors:** Somashekhargouda Patil, C. S. Rudragouda

**Affiliations:** 1 Division of Plant Physiology, Central Coffee Research Institute, Coffee Research Station Post, Balehonnur, Chikkamagaluru, Karnataka, India; 2 Division of Agronomy, Central Coffee Research Institute, Coffee Research Station Post, Balehonnur, Chikkamagaluru, Karnataka, India

**Keywords:** *Coffea arabica*, *Coffea canephora*, coffee agroforestry, coffee holobiont, microbiome engineering, multitrophic interactions, phytobiome, plant physiology

## Abstract

Coffee (*Coffea arabica* L. and *Coffea canephora* Pierre ex A. Froehner) is a globally important perennial plantation crop that sustains the livelihoods of millions of smallholder farmers while making substantial contributions to agricultural economies worldwide global agricultural economies. Nevertheless, sustainable coffee production is increasingly constrained by climate change, declining soil fertility, emerging pests and diseases and the environmental costs associated with intensive use of synthetic agrochemicals. Recent advances in plant microbiome research have transformed the understanding of coffee from an individual organism to a holobiont, where the host plant and its associated microorganisms operate as an integrated biological system. The coffee phytobiome encompasses diverse microbial communities inhabiting the rhizosphere, rhizoplane, endosphere, phyllosphere, anthosphere, carposphere and spermosphere, together with complex multitrophic interactions involving shade trees, soil fauna, insects and the surrounding environment. These interactions collectively regulate nutrient acquisition, carbon assimilation, water-use efficiency, hormonal balance, stress tolerance, immune responses and overall plant productivity. This review critically examines current advances in multitrophic plant–microbe interactions that influence physiological adaptation in coffee, with particular emphasis on sustainable production under Indian agroecological conditions. It highlights the functional functions of plant growth-promoting rhizobacteria (PGPR) and arbuscular mycorrhizal fungi (AMF), endophytic microorganisms and other beneficial microbes in enhancing root development, nutrient cycling, and resilience to biotic and abiotic stresses. Furthermore, the review evaluates recent progress in metagenomics, metatranscriptomics, metabolomics and microbiome engineering for harnessing indigenous microbial resources. Finally, it outlines future research priorities integrating plant physiology, microbial ecology, systems biology, and precision agriculture to develop climate-resilient, resource-efficient, and environmentally sustainable coffee production systems.

## Graphical abstract

**Figure:**
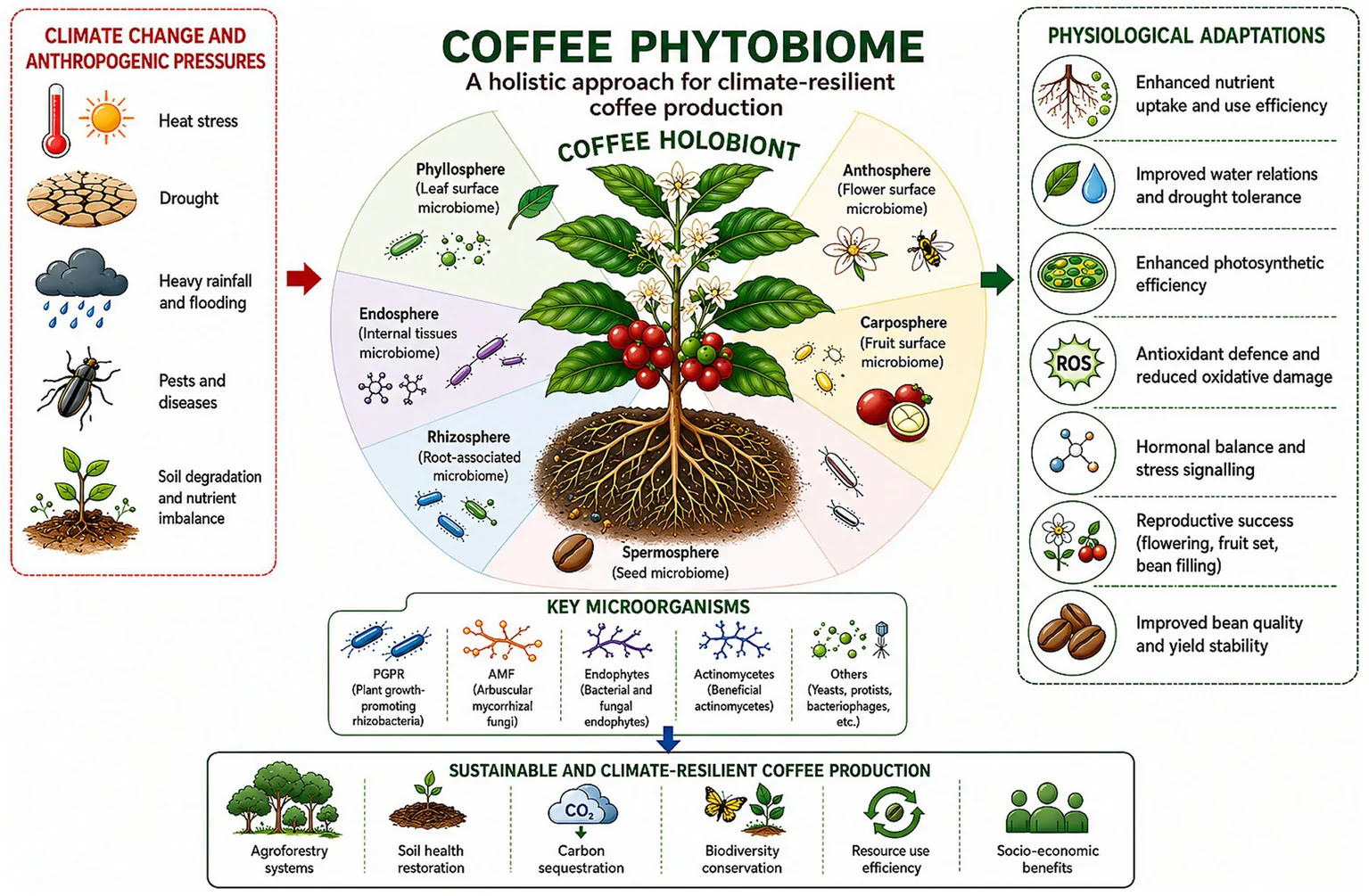

## Highlights

- Coffee functions as a holobiont, where multitrophic interactions among plants, microorganisms, soil biota and shade trees regulate physiological performance and ecosystem resilience.
- Beneficial microorganisms enhance nutrient acquisition, photosynthesis, water-use efficiency, hormonal homeostasis and antioxidant defense, thereby improving coffee adaptation to abiotic and biotic stresses.
- Root exudates, microbial signaling molecules and plant immune pathways coordinate the recruitment and functioning of the coffee phytobiome under changing environmental conditions.
- Multi-omics approaches, microbiome engineering and precision agriculture provide new opportunities for exploiting indigenous microbial resources to improve sustainable coffee production.
- Integrating plant physiology with microbial ecology offers a promising framework for developing climate-resilient coffee agroforestry systems in India.

## 1 Introduction

Coffee an important perennial plantation crops cultivated worldwide supporting the livelihoods of more than 125 million people and contributing substantially to the economies of tropical countries through employment, export earnings and ecosystem services (International Coffee Organization (ICO), 2024). The global coffee industry is dominated by *Coffea arabica* L. and *Coffea canephora* Pierre ex A. Froehner, which together account for more than 99% of commercial coffee production (Davis et al., 2019). India ranks as the world’s sixth-largest coffee-producing country globally and the fifth-largest exporter, cultivating approximately 4.8 lakh hectares of coffee, predominantly under multispecies shade canopies in the Western Ghats biodiversity hotspot (Coffee Board of India, 2024). Unlike intensive monoculture systems, Indian coffee is grown within diverse agroforestry landscapes where coffee plants coexist with native shade trees, soil microorganisms, insects, wildlife and associated vegetation, creating highly complex ecological networks (Bhagwat et al., 2008; Jose, 2009).

Climate change is increasingly disrupting these ecological networks and has emerged as one of the greatest threats to sustainable coffee production. Increasing temperatures, irregular rainfall patterns, prolonged droughts, intense precipitation events and the greater frequency of climatic extremes have altered flowering behavior, reproductive success, berry development and bean quality across major coffee-growing regions (Bunn et al., 2015; DaMatta et al., 2019; IPCC, 2023). In India, irregular blossom and backing showers, prolonged dry periods and heavy monsoon rainfall frequently lead to poor fruit set, nutrient losses, physiological stress and increased incidences of white stem borer (*Xylotrechus quadripes*), coffee berry borer (*Hypothenemus hampei*), coffee leaf rust (*Hemileia vastatrix*) and berry diseases, ultimately reducing yield stability and beverage quality (Jha et al., 2014; Coffee Board of India, 2024; Avelino et al., 2015).

Conventional coffee management has largely relied on chemical fertilizers, pesticides and irrigation to sustain productivity. Although these practices have improved yields, excessive dependence on external inputs has contributed to declining soil quality, reduced microbial diversity, nutrient imbalances and environmental degradation (Tilman et al., 2002; Trivedi et al., 2020). Consequently, sustainable coffee production increasingly requires ecological approaches that enhance crop resilience while minimizing environmental impacts. Among these, the phytobiome has emerged as an integrative framework that considers plant and its associated biological communities influencing growth, adaptation and productivity (Leach et al., 2017).

Phytobiome concept extends beyond traditional plant microbiome by incorporating not only bacteria, fungi, archaea and viruses associated with plants, but also insects, nematodes, soil fauna, neighboring vegetation, climatic conditions and management practices that collectively influence plant performance (Leach et al., 2017; Vandenkoornhuyse et al., 2015). Within this framework, coffee functions as a holobiont, where the host plant and its associated microbial communities collectively determine nutrient acquisition, physiological adaptation, defense responses and reproductive performance. Rather than acting independently, microorganisms continuously exchange metabolites, signaling molecules and nutrients with the host, thereby regulating multiple physiological processes throughout the crop life cycle (Compant et al., 2019; Trivedi et al., 2020).

Coffee-associated microbial communities inhabit diverse ecological niches, including the rhizosphere, rhizoplane, root endosphere, stem endosphere, phyllosphere, anthosphere, carposphere and spermosphere (Vega et al., 2010; de Sousa et al., 2022). These communities comprise plant growth-promoting rhizobacteria, arbuscular mycorrhizal fungi, endophytic fungi, yeasts, actinomycetes and other beneficial microorganisms that participate in biological nitrogen fixation, phosphorus solubilization, micronutrient mobilization, phytohormone biosynthesis, antioxidant regulation and biological control of pathogens (Begum et al., 2019; Ramirez-Lopez et al., 2025). Increasing evidence indicates that these microorganisms regulate root system architecture, photosynthetic efficiency, stomatal conductance, water-use efficiency, osmotic adjustment, flowering intensity and berry development, demonstrating that coffee physiology is strongly influenced by the structure and function of its associated phytobiome (DaMatta et al., 2019; Mishra et al., 2021).

Recent developments in high-throughput sequencing and multi-omics approaches, including metagenomics, metatranscriptomics, metabolomics and culturomics, have substantially advanced our understanding of plant–microbe interactions by revealing the composition and functional roles of complex microbial communities (Trivedi et al., 2020; Compant et al., 2019). However, most coffee microbiome studies remain descriptive, focusing primarily on taxonomic diversity rather than the mechanistic links between microbial functions and physiological adaptation. Relatively few investigations have examined multitrophic interactions in which microorganisms interact simultaneously with coffee plants, shade trees, soil organisms, insects and environmental drivers to determine ecosystem functioning. Although Indian shade-grown coffee agroforestry provides a particularly relevant ecological context for this discussion, the present review addresses coffee phytobiome processes more rapidly across coffee producing regions and uses India as an illustrative case for translation of indigenous microbial resources into climate-resilient production.

Accordingly, this review synthesizes current research on multitrophic plant–microbe interactions within the coffee phytobiome, emphasizing their roles in physiological adaptation, molecular signaling and ecological resilience. Particular attention is given to nutrient acquisition, photosynthesis, water relations, hormonal regulation, antioxidant defense and reproductive development, together with emerging opportunities in microbiome engineering, multi-omics and precision agriculture. Evidence from coffee systems worldwide is integrated with the distinctive ecological setting of Indian coffee agroforestry to identify knowledge gaps and research priorities for phytobiome-based strategies that can enhance sustainable and climate-resilient coffee production.

## 2 Coffee phytobiome and multitrophic plant–microbe interactions

### 2.1 Coffee as a holobiont

The concept of the plant holobiont has fundamentally changed the understanding of plant biology by demonstrating that plants function as integrated biological systems together with their associated microorganisms rather than as autonomous organisms (Vandenkoornhuyse et al., 2015; Trivedi et al., 2020). Coffee plant and its associated microbial communities collectively determine plant growth, physiological adaptation, nutrient acquisition, defense responses and productivity. The collective genetic complement of the host and its associated microorganisms, termed the hologenome, provide functional traits that cannot be explained solely by the plant genome (Leach et al., 2017; Vandenkoornhuyse et al., 2015).

In coffee, the phytobiome comprises microorganisms associated with the rhizosphere, rhizoplane, endosphere, phyllosphere, anthosphere, carposphere and spermosphere, together with shade trees, litter-decomposing organisms, soil fauna, insects and environmental drivers viz., rainfall, temperature and soil physicochemical attributes (Vega et al., 2010; de Sousa et al., 2022). These components interact continuously through nutrient exchange, chemical signaling and ecological feedbacks that regulate physiological performance. Consequently, coffee productivity should be regarded as an outcome arising from the entire biological system rather than the host genotype alone.

Indian coffee agroforestry provides an excellent model for studying holobiont function because coffee is cultivated beneath heterogeneous shade canopies containing numerous native and introduced tree species. These systems create diverse microhabitats that support rich microbial communities and multitrophic interactions, contributing to nutrient cycling, biological pest regulation and ecosystem resilience (Bhagwat et al., 2008; Jose, 2009).

### 2.2 Microbial niches associated with coffee

The coffee phytobiome is spatially organized into several interconnected microbial habitats. Each compartment differs in microbial composition, ecological function and interaction with host physiology.

#### 2.2.1 Rhizosphere

The rhizosphere is the soil region immediately surrounding roots that is shaped by root exudates and represents the most biologically active compartment of the coffee phytobiome (Bulgarelli et al., 2013). Coffee roots release carbohydrates, amino acids, organic acids, flavonoids and phenolic compounds that selectively recruit beneficial microorganisms while simultaneously influencing nutrient availability and soil enzyme activity (Bais et al., 2006; Sasse et al., 2018; Badri and Vivanco, 2009). Rhizosphere microorganisms participate in various biological fixation of nitrogen, solubilization of phosphate, mobilization of potassium, siderophore-mediated iron acquisition and the control of soil-borne pathogens biologically.

#### 2.2.2 Rhizoplane

The rhizoplane constitutes the immediate root surface where microbial attachment and biofilm formation occur. Successful colonization of this compartment enables microorganisms to compete effectively with resident microbiota while facilitating efficient exchange of metabolites and signaling molecules (Compant et al., 2019).

#### 2.2.3 Root endosphere

The endosphere consists of microorganisms that inhabit internal plant tissues without inducing disease. Endophytic bacteria and fungi contribute to nutrient acquisition, antioxidant regulation, phytohormone synthesis and induced systemic resistance while remaining protected from external environmental fluctuations (Hardoim et al., 2015).

#### 2.2.4 Phyllosphere

Leaves constitute one of the most extensive habitats for microbial colonization in terrestrial ecosystems in terrestrial ecosystems. Coffee phyllosphere communities are dominated by bacteria belonging to *Pseudomonas*, *Methylobacterium*, *Sphingomonas* and *Bacillus*, together with diverse yeasts and filamentous fungi (Lindow and Brandl, 2003). These microorganisms contribute to pathogen suppression, ultraviolet protection and modulation of leaf physiological processes.

#### 2.2.5 Anthosphere, carposphere and spermosphere

Flowers, fruits and seeds also harbor specialized microbial communities. Flower-associated microorganisms influence nectar chemistry and pollinator behavior, whereas fruit-associated microbes participate in carbohydrate metabolism and fermentation processes that affect coffee flavor and beverage quality (De Bruyn et al., 2017). Seed-associated microorganisms contribute to vertical transmission of beneficial microbes and early seedling establishment (Truyens et al., 2014).

### 2.3 Major microbial groups in the coffee phytobiome

Coffee-associated microbial communities encompass bacteria, fungi, archaea, viruses and protists, each contributing distinct ecological functions.

Among bacteria, members of the genera *Bacillus*, *Pseudomonas*, *Azospirillum*, *Burkholderia*, *Rhizobium*, *Paenibacillus*, *Enterobacter* and *Streptomyces* are frequently reported as dominant plant growth-promoting taxa (Ramirez-Lopez et al., 2025; Barka et al., 2006). These microorganisms improve plant nutrient acquisition by facilitating nitrogen fixation biologically, solubilization of phosphate, mobilization of potassium and siderophore-mediated iron uptake production while simultaneously synthesizing phytohormones that regulate root growth (Glick, 2014).

Fungal communities are primarily represented by arbuscular mycorrhizal fungi, *Trichoderma*, *Penicillium*, *Aspergillus* and numerous endophytic taxa (Begum et al., 2019). Mycorrhizal fungi increase phosphorus uptake, improve water acquisition and enhance drought tolerance through extensive extraradical hyphal networks (Smith and Read, 2008). *Trichoderma* spp. contribute to biological control by producing antifungal metabolites, hydrolytic enzymes and elicitors that activate host defense pathways (Saxena et al., 2025).

Although comparatively less studied, ammonia-oxidizing archaea participate in nitrogen transformations within acidic coffee soils, while protists regulate bacterial community composition through microbial grazing (Fuchs et al., 2012). Bacteriophages and mycoviruses further influence microbial community dynamics by modulating population structure highlighting multitrophic interactions within the coffee phytobiome.

### 2.4 Multitrophic interactions in coffee agroforestry

Unlike simplified agricultural systems, coffee agroforestry is characterized by interactions among plants, microorganisms, arthropods, vertebrates and environmental drivers operating across multiple trophic levels. Shade trees contribute organic matter through litterfall and root turnover, increasing soil organic carbon and stimulating microbial activity (Jose, 2009). Earthworms, termites and other soil fauna accelerate decomposition, redistribute nutrients and improve soil aggregation, thereby indirectly influencing microbial habitats (Lavelle et al., 2006).

Beneficial microorganisms also interact with pollinators and natural enemies of pests. Nectar-associated yeasts modify floral volatile compounds that affect pollinator visitation, while microbial endophytes can influence herbivore performance by altering plant secondary metabolism (Vannette, 2020). Such multitrophic interactions contribute to ecosystem services extending beyond plant nutrition, including biological pest regulation, pollination and biodiversity conservation.

The diversity of shade-tree species further modifies the coffee phytobiome by altering microclimate, litter quality and root exudation patterns. Comparative studies indicate that agroforestry systems generally support higher microbial diversity and greater functional redundancy than low-shade monocultures, enhancing ecosystem stability under climatic stress (Bhagwat et al., 2008; Vaast et al., 2016).

### 2.5 Factors regulating coffee phytobiome assembly

Assembly of the coffee phytobiome is governed by interactions among host genotype, soil properties, climate and management practices. Coffee genotype influences root exudates composition and therefore the recruitment of specific microbial taxa (de Sousa et al., 2022). Soil pH, organic matter content, moisture and nutrient availability further determine microbial diversity and metabolic activity (Bulgarelli et al., 2013). Elevation and shade intensity modify soil temperature and moisture, creating distinct ecological niches that influence microbial succession (Nesper et al., 2017).

Agronomic interventions such as fertilizer application, irrigation, pruning and pesticide use also alter microbial community composition. Excessive chemical inputs may reduce microbial diversity and suppress beneficial symbionts, whereas organic amendments increase microbial biomass, enzymatic activity and functional diversity (Trivedi et al., 2020). Understanding these interacting drivers essential for developing management strategies promote resilient phytobiomes capable of sustaining coffee productivity under changing environmental conditions (Table 1).

**Table 1:** Major microbial groups associated with coffee and their principal ecological functions.

Microbial group	Representative genera	Major physiological and ecological functions	Coffee specific references
Plant growth-promoting bacteria	*Bacillus*, *Pseudomonas*, *Azospirillum*, *Burkholderia*, *Rhizobium*	Nitrogen fixation, phosphorus solubilization, phytohormone production, siderophore production, disease suppression	Ramirez-Lopez et al. (2025), Muleta et al. (2013), Suriani et al. (2025), and Caldwell et al. (2015)
Actinomycetes	*Streptomyces*	Antibiotic production, pathogen suppression, decomposition of organic matter	Vega et al. (2010) and Ramirez-Lopez et al. (2025)
Arbuscular mycorrhizal fungi	*Rhizophagus*, *Glomus*, *Funneliformis*	Phosphorus uptake, water acquisition, drought tolerance, soil aggregation	Habte and Byappanahalli (1994) and Begum et al. (2019)
Endophytic fungi	*Trichoderma*, *Penicillium*, *Cladosporium*	Antioxidant induction, disease resistance, plant growth promotion	Vega et al. (2010); Asad et al. (2023)
Yeasts	*Metschnikowia*, *Pichia*	Fermentation, modulation of floral and fruit microbiomes	De Bruyn et al. (2017)
Archaea	Ammonia-oxidizing archaea	Nitrogen cycling	Caldwell et al. (2015)
Protists	Cercozoa and amoebae	Regulation of bacterial populations and nutrient mineralization	Coffee-specific evidence limited

Coffee-associated PGPB, particularly members of *Bacillus* and *Pseudomonas*, have been reported to exhibit traits related to nutrient mobilization, phytohormone production and plant growth promotion, while *Bacillus*, *Burkholderia* and *Streptomyces* endophytes isolated directly from coffee have demonstrated PGP and biocontrol potential (Duong et al., 2021; Asad et al., 2023; Ramirez-Lopez et al., 2025).

## 3 Physiological adaptations mediated by the coffee phytobiome

Coffee productivity depends wholly on physiological homeostasis under continuously changing environmental conditions. The coffee phytobiome contributes significantly to this adaptive capacity by regulating nutrient acquisition, photosynthetic efficiency, water relations, hormonal signaling, antioxidant metabolism and reproductive development. Rather than acting independently, beneficial microorganisms function as physiological partners that expand the functional phenotype of the coffee plant through coordinated metabolic and signaling networks. Consequently, physiological adaptation in coffee should be considered a systems-level response emerging from interactions among host plant, microbial communities and weather factors (Compant et al., 2019; Trivedi et al., 2020).

### 3.1 Root system architecture and nutrient acquisition

The root system represents the principal interface between coffee plants and soil microorganisms. Fine roots continuously release carbohydrates, amino acids, phenolics and organic acids that shape rhizosphere microbial communities through selective recruitment processes (Bais et al., 2006; Sasse et al., 2018). These exudates create nutrient-rich microenvironments that favor colonization by plant growth-promoting rhizobacteria (PGPR), arbuscular mycorrhizal fungi (AMF) and endophytic microorganisms that improve nutrient acquisition andenhance tolerance to environmental stresses.

Coffee root morphology exhibits considerable plasticity under varying environmental conditions. Under water-limited environments, plants increase root depth and modify lateral root architecture, thereby improving access to deeper soil moisture while simultaneously altering rhizosphere microbial composition (DaMatta et al., 2019). Beneficial microorganisms reinforce these morphological responses through phytohormone production, particularly indole-3-acetic acid (IAA), cytokinins and gibberellins, which stimulate formation of lateral roots, development root hair and surface area adoptive (Spaepen et al., 2007; Glick, 2014).

Nitrogen remains the most limiting nutrient in many coffee-growing soils. Diazotrophic bacteria such as *Azospirillum*, *Herbaspirillum*, *Burkholderia* and *Rhizobium* contribute biologically fixed nitrogen that supplements soil nitrogen availability, particularly in low-input agroforestry systems (Baldani et al., 2014; Ramirez-Lopez et al., 2025). Likewise, phosphate-solubilizing bacteria including *Bacillus*, *Pseudomonas* and *Enterobacter* mobilize insoluble phosphorus via organic acids secretion and phosphatases, improving availability of phosphorus in acidic tropical soils (Richardson et al., 2009; Richardson and Simpson, 2011).

Arbuscular mycorrhizal fungi represent one of the most influential microbial partners in coffee. Their extensive extraradical hyphal networks increase absorptive area of roots several-fold, enhancing phosphorus uptake, micronutrient acquisition and water absorption while simultaneously improving soil aggregation through glomalin production (Smith and Read, 2008; Rillig, 2004). Coffee seedlings colonized by AMF consistently exhibit higher biomass accumulation, chlorophyll content and nutrient-use efficiency than non-mycorrhizal plants (Habte and Byappanahalli, 1994; Calvino et al., 2010; Beer et al., 1998).

Collectively, these microbial interactions transform the coffee root system into a highly efficient nutrient acquisition network capable of sustaining plant growth under nutrient-poor tropical soils.

### 3.2 Regulation of photosynthesis and carbon assimilation

Photosynthesis is the primary physiological process determining coffee productivity because it supplies the carbohydrates required for vegetative growth, flowering and bean development. Microbial communities influence photosynthesis both directly and indirectly through improved nutrient acquisition, hormonal regulation, maintenance of chlorophyll content and oxidative damage mitigation (Compant et al., 2019; Mishra et al., 2021).

Nitrogen-fixing and phosphate-solubilizing microorganisms enhance chlorophyll biosynthesis by increasing the nitrogen availability, magnesium and phosphorus, are essential components of the photosynthetic apparatus (Richardson et al., 2009). Enhanced nutrient availability promotes Rubisco synthesis, chloroplast development and ATP production, ultimately increasing net photosynthetic rate (Pn), stomatal conductance (gs) and mesophyll efficiency (DaMatta et al., 2007).

AMF further improve photosynthesis by maintaining leaf phosphorus concentrations and facilitating efficient ATP-dependent carbon fixation under nutrient-limited conditions (Smith and Read, 2008). In coffee, increased mycorrhizal colonization has been associated with greater chlorophyll concentration, higher quantum efficiency of photosystem II (Fv/Fm) and improved electron transport rates, particularly under moderate drought stress (Begum et al., 2019).

Recent evidence indicates that beneficial microorganisms also regulate photosynthetic performance through modulation of phytohormone balance. Cytokinins synthesized by rhizobacteria delay chlorophyll degradation, whereas microbial production of ACC deaminase reduces stress-induced ethylene accumulation, thereby preventing premature senescence of photosynthetically active leaves (Glick, 2014). These interactions collectively maintain carbon assimilation during environmental stress, increasing the supply of assimilates available for reproductive development.

### 3.3 Water relations and drought adaptation

Water availability is among the most critical determinants of coffee physiology, particularly during flowering, berry expansion and bean filling. Leaf water potential, stomatal conductance, photosynthesis and reproductive success reduced by drought, ultimately limiting yield (DaMatta et al., 2019). Beneficial microorganisms substantially improve drought tolerance by modifying root hydraulic properties, osmotic adjustment and antioxidant metabolism.

Coffee plants respond to water deficit through coordinated adjustments in stomatal regulation, photosynthetic metabolism, osmotic balance and primary metabolism, with substantial genotypic differences in drought tolerance (Menezes-Silva et al., 2017; Chekol et al., 2024). However, direct experimental evidence linking specific members of the coffee phytobiome to these physiological drought responses remains comparatively limited (Ramirez-Lopez et al., 2025; Wei et al., 2026).

AMF enhance hydraulic conductivity through extensive hyphal networks that increase access to soil water beyond the root depletion zone (Begum et al., 2019). Simultaneously, PGPR stimulate root proliferation and increase aquaporin expression, facilitating more efficient water uptake (Vurukonda et al., 2016). Improved root hydraulic conductance enables coffee plants to maintain higher relative water content (RWC), stomatal function and photosynthetic activity during moderate drought.

Microbial inoculation also promotes compatible osmolytes accumulation including proline, glycine betaine and soluble sugars, thereby maintaining cellular turgor under declining soil moisture (Mishra et al., 2021). Enhanced water-use efficiency results from coordinated regulation of stomatal aperture, improved carbon assimilation and reduced transpirational water loss.

### 3.4 Hormonal regulation of physiological adaptation

Plant-associated microorganisms influence coffee physiology through biosynthesis or modulation of plant hormones. Microbial production of indole-3-acetic acid stimulates root branching and root hair development, whereas cytokinins regulate shoot growth and chloroplast maintenance (Spaepen et al., 2007).

Abscisic acid (ABA) plays a central role in drought responses by regulating stomatal closure. Increasing evidence suggests that beneficial microorganisms modify ABA signaling, enabling more efficient stomatal regulation while maintaining photosynthetic performance (Vurukonda et al., 2016). Microbial ACC deaminase lowers ethylene accumulation under stress, preventing inhibition of root growth and promoting continued nutrient acquisition (Glick, 2014).

Cross-talk among auxins, ABA, jasmonic acid (JA) and salicylic acid (SA) integrates developmental and defense responses, allowing coffee plants to balance growth with resistance against pathogens and environmental stress (Pieterse et al., 2014) (Table 2).

**Table 2:** Physiological processes regulated by beneficial microorganisms in coffee.

Physiological process	Beneficial microorganisms	Mechanism	Plant response
Root development	*Bacillus*, *Pseudomonas*, *Azospirillum*	IAA production	Increased root biomass
Nitrogen uptake	Diazotrophic bacteria	Biological N fixation	Higher chlorophyll content
Phosphorus uptake	AMF, *Bacillus*	Hyphal transport, phosphate solubilization	Improved ATP synthesis
Photosynthesis	PGPR + AMF	Nutrient acquisition, cytokinin production	Higher photosynthetic rate
Water relations	AMF, PGPR	Aquaporin regulation, osmotic adjustment	Higher WUE
Hormonal regulation	PGPR	ACC deaminase, IAA	Reduced stress ethylene
Antioxidant defense	Endophytes, *Trichoderma*	Enhanced SOD, CAT, APX	Reduced oxidative damage
Reproductive development	PGPR, AMF	Carbon allocation, hormone balance	Better flowering and fruit retention

## 4 Molecular mechanisms underpinning coffee–microbe interactions

### 4.1 Root exudate-mediated recruitment of the coffee phytobiome

Plant roots continuously secrete a primary and secondary metabolites that shape the composition and activity of the rhizosphere microbiome. These root exudates function as nutritional resources and signaling molecules, selectively recruiting microorganisms capable of establishing beneficial associations with the host plant (Bais et al., 2006; Sasse et al., 2018). In coffee, fine roots release carbohydrates, amino acids, organic acids, flavonoids, phenolics and alkaloids such as caffeine, generating distinct chemical gradients that influence microbial colonization and community assembly (de Sousa et al., 2022; Berendsen et al., 2012).

Recent metabolomic studies suggest that environmental conditions strongly influence coffee root exudation patterns. Drought, nutrient deficiency and pathogen attack modify the concentration and composition of exuded metabolites, thereby altering microbial recruitment and functional diversity (Hoseinzadeh et al., 2020). Organic acids including citrate and malate enhance phosphate-solubilizing bacterial populations by increasing phosphorus availability in acidic soils, whereas amino acids and simple sugars promote colonization by plant growth-promoting rhizobacteria (Richardson et al., 2009; de Vries et al., 2018). Coffee-specific metabolites, particularly chlorogenic acids and caffeine, may further shape microbial communities because several rhizosphere bacteria possess metabolic pathways capable of degrading methylxanthines and phenolic compounds (Summers et al., 2012). Nevertheless, the composition of coffee root exudates across developmental stages and environmental conditions remains poorly characterized, representing an important research priority.

Root exudates also mediate communication with arbuscular mycorrhizal fungi (AMF). Strigolactones released from roots stimulate hyphal branching and facilitate the establishment of mycorrhizal symbiosis, thereby enhancing phosphorus acquisition and water uptake (Akiyama et al., 2005). Such interactions highlight the dynamic role of root exudation in coordinating multitrophic interactions between coffee plants, beneficial fungi and rhizosphere bacteria.

### 4.2 Microbial recognition and plant immune signaling

Successful colonization of coffee roots requires precise molecular communication between host cells and microorganisms. Plants detect conserved microbial molecules known as microbe-associated molecular patterns (MAMPs) via plasma membrane-localized pattern recognition receptors (PRRs), triggering pattern-triggered immunity (PTI) (Boller and Felix, 2009). Typical MAMPs include bacterial flagellin, lipopolysaccharides, peptidoglycans and fungal chitin fragments.

Activation of PTI initiates a cascade of intracellular events involving calcium influx, reactive oxygen species (ROS) production, mitogen-activated protein kinase (MAPK) signaling and transcriptional reprogramming that collectively enhance plant defense (Jones and Dangl, 2006). Beneficial microorganisms have evolved strategies to attenuate excessive immune activation while maintaining mutualistic interactions. Many PGPR suppress local defense responses through secretion of effector proteins, extracellular polysaccharides or phytohormones, enabling stable colonization without compromising plant immunity (Pieterse et al., 2014; Bhattacharyya and Jha, 2012).

When pathogens deliver virulence effectors into host cells, intracellular resistance proteins recognize these molecules and activate effector-triggered immunity (ETI), resulting in stronger defense responses that often include hypersensitive cell death (Jones and Dangl, 2006). Although PTI and ETI have been extensively investigated in model plants, comparable studies in coffee remain limited, highlighting the need for functional genomic analyses of coffee immune signaling.

### 4.3 Induced systemic resistance and systemic acquired resistance

Beneficial microorganisms enhance coffee defense not only through direct antagonism against pathogens but also by priming systemic immune responses. Two major forms of induced resistance have been described: induced systemic resistance (ISR) and systemic acquired resistance (SAR) (Pieterse et al., 2014; Busby et al., 2017).

ISR is typically activated by PGPR and certain beneficial fungi through jasmonic acid (JA) and ethylene signaling pathways. Colonized roots generate systemic signals that increase defense readiness throughout the plant without imposing substantial metabolic costs under non-stress conditions (Pieterse et al., 2014). In coffee, ISR may enhance resistance against foliar pathogens such as coffee leaf rust (*Hemileia vastatrix*) and anthracnose caused by *Colletotrichum* spp., although mechanistic studies remain scarce.

In contrast, SAR is generally induced following pathogen infection and depends largely on salicylic acid (SA) signaling. Activation of SAR results in accumulation of pathogenesis-related proteins, antimicrobial metabolites and long-lasting systemic immunity (Durrant and Dong, 2004). Emerging evidence indicates that beneficial microorganisms can modulate both ISR and SAR simultaneously, enabling coffee plants to maintain balanced growth and defense under fluctuating environmental conditions.

### 4.4 Quorum sensing and biofilm formation

Communication among microbial populations is coordinated through quorum sensing, a density-dependent signaling mechanism mediated by small diffusible molecules including N-acyl homoserine lactones in Gram-negative bacteria and oligopeptides in Gram-positive bacteria (Papenfort and Bassler, 2016; Cannell, 1985). Quorum sensing regulates biofilm formation, motility, antibiotic production and expression of plant growth-promoting traits.

Biofilms formed on coffee root surfaces provide structural stability, facilitate nutrient exchange and protect microbial communities against environmental stress (Compant et al., 2019). Beneficial biofilms also create physical barriers that limit pathogen colonization while increasing persistence of microbial inoculants in the rhizosphere. Understanding quorum sensing mechanisms may therefore improve the design of synthetic microbial consortia for sustainable coffee production.

### 4.5 Microbial metabolites regulating coffee physiology

Beneficial microorganisms produce diverse metabolites that directly influence plant physiology (Table 3). Phytohormones including indole-3-acetic acid (IAA), gibberellins and cytokinins regulate root architecture, shoot development and chloroplast maintenance (Spaepen et al., 2007; Carrion et al., 2019). ACC deaminase lowers stress-induced ethylene concentrations, thereby sustaining root elongation under drought and nutrient deficiency (Glick, 2014).

**Table 3:** Principal molecular mechanisms governing coffee–microbe interactions.

Mechanism	Major molecules/Components	Physiological significance
Root exudation	Sugars, amino acids, organic acids, phenolics, caffeine	Microbial recruitment and nutrient mobilization
Pattern-triggered immunity (PTI)	Flagellin, chitin, PRRs	Early defense activation
Effector-triggered immunity (ETI)	Resistance proteins, pathogen effectors	Strong pathogen resistance
Induced systemic resistance (ISR)	Jasmonic acid, ethylene	Enhanced defense priming
Systemic acquired resistance (SAR)	Salicylic acid, PR proteins	Long-term systemic immunity
Quorum sensing	N-acyl homoserine lactones, peptides	Biofilm formation and microbial coordination
Phytohormone production	IAA, cytokinins, gibberellins	Root growth and photosynthesis
ACC deaminase	ACC deaminase enzyme	Reduced stress ethylene
Siderophores	Ferric ion chelators	Iron acquisition and pathogen suppression
VOCs	Alcohols, ketones, terpenoids	Growth promotion and systemic signaling

Siderophores enhance iron acquisition by chelating ferric ions, while lipopeptides and antibiotics suppress soil-borne pathogens (Miethke and Marahiel, 2007). Volatile organic compounds (VOCs) released by rhizobacteria stimulate photosynthesis, improve biomass accumulation and activate systemic resistance even without direct root colonization (Ryu et al., 2003). Exopolysaccharides contribute to aggregation of soil, retention of water and microbial persistence, particularly under conditions of drought (Vurukonda et al., 2016).

Together, these metabolites establish an extensive biochemical communication network that integrates microbial metabolism with coffee physiological responses.

### 4.6 Multi-omics approaches reveal functional interactions

Recent advances in multi-omics technologies have revolutionized phytobiome research by enabling simultaneous characterization of microbial diversity, gene expression, protein abundance and metabolite production (Trivedi et al., 2020; Dussert et al., 2025). Metagenomics identifies microbial taxonomic composition and functional genes, whereas metatranscriptomics reveals actively expressed metabolic pathways. Metabolomics characterizes chemical interactions between coffee roots and microorganisms, while proteomics provides insights into stress-responsive proteins involved in symbiosis and defense.

Integration of these approaches allows researchers to identify key microbial functions associated with desirable physiological traits such as improved photosynthesis, enhanced nutrient-use efficiency or drought tolerance. However, multi-omics studies remain rare in coffee, particularly under field conditions. Future investigations integrating omics technologies with physiological phenotyping and ecological analyses will provide a systems-level understanding of coffee phytobiome function.

## 5 Coffee phytobiome-mediated climate resilience

Climate change is one of the greatest threats to global coffee production, affecting both yield and bean quality through increasing temperatures, irregular precipitation, prolonged drought, and extreme weather events (Bunn et al., 2015; DaMatta et al., 2019; IPCC, 2023). Arabica coffee is particularly vulnerable because its optimum temperature range (18–23 °C) is narrow, whereas Robusta exhibits relatively greater tolerance to elevated temperatures and water deficits (DaMatta and Ramalho, 2006; Fierer, 2017). Climatic stress disrupts photosynthesis, flowering, fruit development, nutrient uptake and carbon allocation, thereby reducing productivity and increasing susceptibility to pests and diseases.

The coffee phytobiome provides an additional adaptive layer that enhances plant resilience through improved nutrient acquisition, maintenance of water status, antioxidant regulation, immune priming and stabilization of soil ecosystem functions (Compant et al., 2019; Trivedi et al., 2020). Rather than acting independently, microorganisms and their host coordinate physiological responses that enable coffee plants to tolerate environmental stress while maintaining metabolic activity.

### 5.1 Heat stress adaptation

Increasing air temperatures negatively affect coffee by reducing pollen viability, accelerating respiration, destabilizing photosystem II, increasing photorespiration and impairing bean filling (DaMatta et al., 2019; Chaparro et al., 2014). Heat stress also enhancesreactive oxygen species (ROS) production, resulting in oxidative damage to chloroplast membranes, proteins and nucleic acids (Hasanuzzaman et al., 2020).

Beneficial microorganisms mitigate heat stress through several complementary mechanisms. Plant growth-promoting rhizobacteria (PGPR) enhance chlorophyll stability, improve stomatal regulation and increase antioxidantenzyme activities including superoxide dismutase (SOD), catalase (CAT) and ascorbate peroxidase (APX), thereby reducing oxidative injury (Vurukonda et al., 2016; Mishra et al., 2021). Endophytic fungi further enhance thermotolerance by inducing heat shock proteins, osmoprotectants and compatible solutes that preserve cellular integrity during thermal stress (Rodriguez et al., 2008).

In coffee, microbial-mediated improvements in photosynthetic efficiency under elevated temperature remain poorly studied, although evidence from other perennial crops suggests that phytobiome-assisted regulation of chlorophyll fluorescence, Rubisco activity and electron transport may contribute substantially to heat resilience (Compant et al., 2019).

### 5.2 Drought tolerance

Drought is among the most critical constraints to coffee production because flowering, berry expansion and bean filling require adequate soil moisture (DaMatta et al., 2019; Tilman et al., 2002). Water deficits reduce stomatal conductance, leaf water potential, carbon assimilation and carbohydrate availability while increasing oxidative stress.

AMF improve drought tolerance through extensive hyphal networks that increase access to soil water beyond the root depletion zone (Smith and Read, 2008). Simultaneously, PGPR enhance root hydraulic conductivity by stimulating root branching and regulating aquaporin expression (Vurukonda et al., 2016). These interactions improve relative water content (RWC), water-use efficiency (WUE) and stomatal regulation while sustaining photosynthetic activity.

Microbial inoculation also promotes osmotic adjustment through increased accumulation of proline, soluble sugars and glycine betaine (Mishra et al., 2021). These compatible solutes maintain cell turgor and membrane stability during prolonged drought. In addition, microorganisms producing ACC deaminase reduce stress-induced ethylene accumulation, preventing premature root senescence and maintaining nutrient uptake (Glick, 2014).

Coffee-specific evidence is beginning to support these mechanisms. Recent synthesis of rhizospheric and endophytic PGPB associated with *C. arabica* and *C. canephora* reports that coffee-derived bacteria can express phosphate solubilization, biological nitrogen fixation, auxin and siderophore production and ACC-deaminase activity, with greenhouse studies showing improved growth and tolerance to drought or nutrient limitation (Ramirez-Lopez et al., 2025). In Robusta coffee, inoculation with *Bacillus nitrificans* and *B. velezensis*, particularly as a consortium, enhanced plant growth, yield and antioxidant-related phytochemical traits while demonstrating root colonization, IAA production, nitrogen fixation and phosphorus solubilization (Suriani et al., 2025). Nevertheless, direct coffee studies quantifying drought physiology such as leaf water potential, stomatal conductance, chlorophyll fluorescence, aquaporin expression and recovery after rewatering remain scarce; therefore, mechanistic claims should be interpreted as emerging rather than fully established in coffee.

### 5.3 Responses to excess rainfall and waterlogging

Climate projections indicate an increase in the frequency of intense rainfall events in many coffee-growing regions (IPCC, 2023). Excess soil moisture limits oxygen diffusion, reduces root respiration and alters microbial community composition, favoring anaerobic microorganisms while suppressing beneficial aerobic taxa (Trivedi et al., 2020).

Beneficial microbial communities may partially alleviate these effects by improving soil aggregation, enhancing drainage and maintaining nutrient cycling following heavy rainfall. AMF contribute to soil structural stability through glomalin production, whereas decomposer microorganisms accelerate recovery of nutrient cycling after flooding events (Rillig, 2004). Nevertheless, prolonged waterlogging remains detrimental to coffee, emphasizing the importance of integrated soil and water management alongside phytobiome-based strategies.

### 5.4 Oxidative stress and antioxidant defense

Oxidative stress represents a common consequence of heat, drought, flooding and pathogen attack. Excess ROS such as superoxide radicals, hydrogen peroxide and hydroxyl radicals damage membranes, proteins and DNA, ultimately impairing photosynthesis and growth (Hasanuzzaman et al., 2020).

Beneficial microorganisms enhance antioxidant capacity by stimulating activities of SOD, CAT, APX and glutathione reductase while increasing concentrations of non-enzymatic antioxidants including ascorbate, glutathione and phenolic compounds (Mishra et al., 2021). Such responses preserve membrane integrity, maintain chloroplast function and improve recovery following environmental stress. Endophytic fungi and *Trichoderma* spp. are particularly effective in activating antioxidant defense pathways while simultaneously priming immune responses against pathogens (Saxena et al., 2025).

### 5.5 Climate resilience in coffee agroforestry

Indian coffee is predominantly cultivated under shade, providing natural microclimatic buffering against climatic extremes. Shade trees reduce maximum canopy temperatures, moderate soil evaporation, increase litter inputs and promote diverse microbial habitats (Bhagwat et al., 2008; Jose, 2009). These conditions favor greater microbial diversity and functional redundancy compared with simplified production systems.

The interaction between shade trees and beneficial microorganisms creates a resilient multitrophic network that improves soil carbon sequestration, nutrient cycling and water retention while reducing physiological stress on coffee plants (Vaast et al., 2016). Consequently, phytobiome-mediated adaptation should be considered within the broader context of agroforestry ecosystem management rather than isolated microbial inoculation (Table 4).

**Table 4:** Climate stress responses mediated by the coffee phytobiome.

Climate stress	Physiological effects on coffee	Phytobiome-mediated responses
Heat	Reduced photosynthesis, ROS accumulation	Antioxidant activation, chlorophyll protection, heat tolerance
Drought	Reduced stomatal conductance, water deficit	Improved WUE, osmotic adjustment, deeper rooting
Heavy rainfall	Nutrient leaching, root hypoxia	Soil aggregation, nutrient recovery, microbial resilience
Nutrient stress	Reduced growth and chlorophyll	Nitrogen fixation, phosphorus solubilization, siderophore production
Oxidative stress	Membrane damage, reduced carbon assimilation	Increased SOD, CAT, APX and phenolic compounds

## 6 Translational approaches for harnessing the coffee phytobiome

### 6.1 Multi-omics for functional characterization of the coffee phytobiome

Traditional culture-dependent methods have provided valuable insights into coffee-associated microorganisms; however, they detect only a limited fraction of microbial diversity because many microorganisms remain unculturable under laboratory conditions (Bulgarelli et al., 2013). Multi-omics technologies have transformed phytobiome research by enabling simultaneous characterization of microbial diversity, functional genes, metabolic pathways and host physiological responses (Trivedi et al., 2020).

Metagenomic sequencing identifies the taxonomic composition and functional potential of microbial communities associated with coffee roots, leaves and fruits (de Sousa et al., 2022). Metatranscriptomics reveals actively expressed microbial genes during nutrient exchange, drought adaptation and pathogen interactions, whereas metaproteomics identifies proteins involved in stress tolerance and microbial colonization (Petrovska et al., 2013). Metabolomics complements these approaches by characterizing root exudates, phytohormones, volatile organic compounds and microbial secondary metabolites that mediate communication between coffee plants and associated microorganisms (Hoseinzadeh et al., 2020).

Complementary datasets integration provides a systems understanding of coffee physiology level by linking microbial functions with measurable plant traits such as photosynthesis, nutrient-use efficiency, chlorophyll fluorescence, water-use efficiency and bean quality. Such integrated analyses remain scarce in coffee but represent one of the most promising research directions for identifying microbial functions that consistently enhance productivity under field conditions.

### 6.2 Synthetic microbial communities and microbiome engineering

Single microbial inoculants often show inconsistent performance under field conditions because introduced microorganisms must compete with established indigenous communities and adapt to varying environmental conditions (Compant et al., 2019). Consequently, recent research has shifted toward the development of synthetic microbial communities (SynComs) comprising carefully selected microorganisms with complementary functional traits.

An ideal coffee SynCom may include nitrogen-fixing bacteria, phosphate-solubilizing bacteria, arbuscular mycorrhizal fungi, endophytic fungi and antagonistic microorganisms that collectively improve nutrient acquisition, stress tolerance and disease suppression. Designing such communities requires consideration of microbial compatibility, functional redundancy and ecological stability rather than simply combining multiple beneficial strains (Vorholt et al., 2017).

Microbiome engineering also encompasses host-mediated microbiome selection, microbiome transplantation and targeted manipulation of root exudates to recruit beneficial indigenous microorganisms (Mueller and Sachs, 2015; Cordovez et al., 2019). For perennial crops such as coffee, where long-term microbial associations are established over several years, microbiome engineering offers considerable potential for enhancing resilience without excessive dependence on chemical inputs.

### 6.3 Artificial intelligence and precision phytobiome management

Artificial intelligence (AI), machine learning and remote sensing technologies are increasingly integrated into precision agriculture for monitoring crop performance and predicting environmental stress. These tools also provide opportunities for precision management of the coffee phytobiome.

Machine-learning algorithms can integrate microbiome profiles, soil properties, climatic variables and physiological measurements to predict nutrient deficiencies, disease outbreaks and stress responses (Jayaramaiah et al., 2024; Hoseinzadeh et al., 2020). Hyperspectral imaging, chlorophyll fluorescence imaging and thermal infrared sensing enable non-destructive assessment of photosynthetic efficiency, canopy temperature and plant water status, facilitating early detection of stress before visible symptoms appear (Mahlein, 2016).

For coffee, combining physiological phenotyping with microbiome information may support the development of decision-support systems that recommend site-specific microbial inoculants, irrigation schedules and nutrient management strategies. Although these technologies are still emerging, they have considerable potential to improve resource-use efficiency and reduce production costs in climate-sensitive coffee landscapes.

### 6.4 Indigenous microbial resources for Indian coffee

Indian coffee plantations, particularly those located in the Western Ghats, harbor exceptionally diverse microbial communities owing to high shade-tree diversity, abundant organic matter and favorable climatic conditions (Bhagwat et al., 2008; Dubey et al., 2019). These indigenous microorganisms represent valuable biological resources that remain largely unexplored.

Developing region-specific microbial inoculants from native coffee ecosystems may offer several advantages over imported commercial products because indigenous microorganisms are already adapted to local soils, climate and coffee genotypes. Systematic isolation, functional characterization and preservation of these microorganisms through national microbial resource banks would facilitate future breeding and bioformulation programs.

Long-term field experiments across major coffee-growing regions, including Chikkamagaluru, Kodagu, Wayanad and the Eastern Ghats, are required to identify microbial consortia capable of consistently enhancing coffee physiology under contrasting environmental conditions.

### 6.5 Challenges and opportunities

Despite rapid advances in phytobiome research, several constraints limit commercial application. Microbial inoculants frequently exhibit inconsistent field performance because of environmental variability, competition with resident microbiota and differences among coffee genotypes. Standardized protocols for inoculum production, formulation, storage and field application remain limited, particularly for perennial crops.

Furthermore, regulatory frameworks governing microbial bioinoculants differ among countries, potentially delaying commercialization of newly developed microbial products. Economic feasibility, farmer awareness and compatibility with existing agronomic practices must also be considered before large-scale implementation.

Nevertheless, increasing availability of multi-omics technologies, advances in microbial culturomics, artificial intelligence and systems biology provide unprecedented opportunities to develop microbiome-informed coffee production systems that enhance productivity while reducing environmental impacts. Critical knowledge gaps remain, however. Coffee studies are still geographically uneven and are dominated by taxonomic surveys or short-term seedling experiments, strain persistence, colonization stability and functional expression under mature field conditions are poorly resolved. Causal links between microbial taxa and coffee physiological traits such as photosynthetic capacity, hydraulic conductance, reproductive success and bean quality require validation through controlled inoculation, gnotobiotic or reductionist systems and multi-location field trials. Greater attention is also needed to genotype × microbiome × environment interactions, including differences between Arabica and Robusta and between shade, elevation, soil and management regimes. Future work should integrate longitudinal multi-omics with physiological phenotyping, define core and stress-responsive microbiomes, establish biosafety and quality-control standards for SynComs and evaluate agronomic consistency and economic returns. For India, these priorities should be pursued within the wider global coffee context rather than as an isolated system, with indigenous microbial resources serving as locally adapted candidates for comparative validation across coffee-growing environments (Table 5).

**Table 5:** Emerging technologies for phytobiome-assisted sustainable coffee production.

Technology	Primary application	Expected benefit
Metagenomics	Microbial diversity and functional genes	Identification of beneficial taxa
Metatranscriptomics	Active microbial pathways	Functional validation
Metabolomics	Root exudates and microbial metabolites	Understanding signaling networks
Synthetic microbial communities	Multi-strain bioinoculants	Stable field performance
AI and machine learning	Stress prediction and decision support	Precision management
Thermal and hyperspectral imaging	Non-destructive physiological monitoring	Early stress detection
Precision irrigation	Site-specific water management	Improved water-use efficiency
Microbiome engineering	Targeted manipulation of microbial communities	Climate resilience

## 7 Conclusions and future perspectives

The coffee phytobiome represents an integral component of plant physiology rather than an external ecological entity. Through coordinated multitrophic interactions involving bacteria, fungi, archaea, protists, soil fauna and shade trees, beneficial microorganisms regulate nutrient acquisition, photosynthesis, water relations, hormonal homeostasis, antioxidant defense and reproductive development. These interactions expand the adaptive capacity of coffee plants under environmental stress and contribute to sustainable productivity.

Recent advances in molecular biology, multi-omics and microbial ecology have substantially improved understanding of coffee-associated microbial communities; however, translation into practical applications remains limited. Future research should prioritize functional validation of microbial traits, integration of physiological phenotyping with omics technologies, development of synthetic microbial communities and long-term field evaluation across diverse coffee-growing environments.

For India, establishing a National Coffee Phytobiome Initiative could accelerate the discovery and deployment of indigenous microbial resources tailored to Arabica- and Robusta-based agroforestry systems. Such an initiative should integrate the expertise of the Coffee Board of India, the Central Coffee Research Institute (CCRI), universities and industry partners to develop climate-resilient microbial technologies. Combining phytobiome science with precision agriculture, artificial intelligence and sustainable agroforestry management offers a promising pathway for enhancing productivity, conserving biodiversity and improving the long-term resilience of coffee production under changing climatic conditions.
